# Development and validation of a machine learning-based model for perioperative stroke prediction in noncardiac, nonvascular, and nonneurosurgical patients

**DOI:** 10.3389/fphys.2025.1624898

**Published:** 2025-06-20

**Authors:** Xuhui Cong, Xuli Zou, Ruilou Zhu, Yubao Li, Lu Liu, Jiaqiang Zhang

**Affiliations:** ^1^ Department of Anesthesia and Perioperative Medicine, Zhengzhou University People’s Hospital and Henan Provincial People’s Hospital, Zhengzhou, Henan, China; ^2^ Xinxiang Medical University, Xinxiang, Henan, China; ^3^ Zhengzhou University, Zhengzhou, Henan, China

**Keywords:** perioperative stroke, machine learning, gradient boosting machine (GBM), noncardiac, nonvascular, and nonneurosurgical procedures, predictive model, clinical decision support

## Abstract

**Introduction:**

Perioperative stroke is a rare but severe complication that significantly impacts postoperative recovery and survival. This study aimed to develop a machine learning-based predictive model for perioperative stroke risk in patients undergoing noncardiac, nonvascular, and nonneurosurgical procedures.

**Methods:**

This retrospective cohort study was conducted using electronic medical records from 106,328 patients at Henan Provincial People’s Hospital, with data from 2,986 patients analyzed. Nine machine learning models were developed to predict perioperative stroke risk, incorporating key variables such as age, history of stroke, comorbidities, surgical factors, and intraoperative data. The models’ performance was evaluated using standard metrics, including area under the receiver operating characteristic curve (AUC), accuracy, sensitivity, specificity, and F1 score.

**Results:**

Among the nine models, the gradient boosting machine (GBM) demonstrated the best performance. In the training set, GBM achieved an AUC of 0.966 (95% CI: 0.957–0.975), with accuracy, sensitivity, specificity, and an F1 score of 90.4%, 90.4%, 81.8%, and 79.0%, respectively. In the validation set, the model maintained strong performance, with an AUC of 0.936 (95% CI: 0.917–0.954), accuracy of 82.6%, sensitivity of 88.8%, specificity of 81.0%, and an F1 score of 67.1%. In comparison, other models, such as logistic regression, support vector machine (SVM), and neural networks, exhibited lower AUC and less favorable performance metrics. Overall, GBM outperformed all models, demonstrating the best balance across accuracy, sensitivity, specificity, and F1 score.

**Conclusion:**

The GBM model demonstrated strong predictive performance and generalizability for perioperative stroke risk in noncardiac, nonvascular, and nonneurosurgical patients. The integration of this model into a real-time clinical decision support system enhances clinical decision-making by enabling the early identification of high-risk patients and facilitating personalized interventions.

## 1 Introduction

Perioperative stroke is a rare yet severe postoperative complication characterized by the sudden onset of neurological impairment during surgery. This condition significantly affects recovery times and long-term survival outcomes (Mashour et al., 2011). Perioperative stroke results from the interplay of preoperative comorbidities, surgical risks, intraoperative hemodynamic fluctuations, and individual physiological factors (Vlisides and Mashour, 2016). Given the serious clinical consequences, improving preoperative risk assessment methods is crucial.

Traditional risk assessment methods, such as static scoring systems (e.g., CHA_2_DS_2_-VASc) (Tasbulak and Sahin, 2022), rely heavily on manually selected variables, including age and hypertension. Although these systems serve as useful tools for initial risk stratification, they have notable limitations. Their linear assumptions fail to account for nonlinear interactions, such as the combined effect of carotid stenosis (>50%) and intraoperative hypotension on stroke risk (Lengyel et al., 2024). Moreover, these methods overlook dynamic intraoperative factors, like fluctuations in the neutrophil-to-lymphocyte ratio, which have been linked to postoperative thrombotic events. These shortcomings undermine their generalizability across different surgical populations, as demonstrated by a study where traditional models failed to predict strokes in cancer patients with hypercoagulable states (Al Mouslmani et al., 2025). Furthermore, the manual selection of features can result in the exclusion of important predictive factors, such as intraoperative glycemic variability, diminishing the model’s robustness.

In contrast, machine learning (ML) offers a promising alternative by processing high-dimensional data and revealing complex, nonlinear relationships. Recent studies have highlighted its superior performance in predicting perioperative strokes (Pfitzner et al., 2021; Abraham et al., 2023). For instance, deep learning models integrating fibrinogen levels and carotid plaque morphology have demonstrated high sensitivity in predicting embolic strokes, outperforming traditional logistic regression models (Jamthikar et al., 2021; Patel et al., 2024). Additionally, reinforcement learning algorithms, which utilize real-time hemodynamic data, have successfully reduced stroke incidence in vascular surgery patients by adapting blood pressure management strategies. However, despite these advancements, research remains fragmented, with limited exploration of cross-specialty applicability and integration of multi-omics data. As a result, perioperative stroke prediction remains inadequately addressed.

This study used real-world clinical data from a large-scale database to develop predictive models for perioperative stroke using various machine learning algorithms. By integrating preoperative assessments, surgical variables, and perioperative events, we aimed to create an early warning system that enhances clinical decision-making and facilitates precision interventions (Kwun et al., 2025).

## 2 Methods

### 2.1 Data source

This retrospective cohort study analyzed electronic medical records of patients treated at Henan Provincial People’s Hospital between November 2014 and June 2021. The database contained comprehensive, high-quality demographic and clinical data, including patient characteristics, comorbidities, and laboratory results, forming a solid basis for analysis. The hospital’s Institutional Review Board approved the study protocol (Approval No. 2021-157), and all procedures complied with the Declaration of Helsinki. The ethics committee waived the need for informed consent due to the use of anonymized data and adherence to ethical standards.

#### 2.1.1 Inclusion criteria

1. Age ≥18 years, undergoing noncardiac, nonvascular, and nonneurosurgical procedures under general anesthesia.
2. American Society of Anesthesiologists (ASA) physical status classification I–III.
3. Availability of complete perioperative clinical and laboratory data.
4. At least one postoperative follow-up within 30 days.

#### 2.1.2 Exclusion criteria

1. Patients undergoing cardiac, major vascular, or neurosurgical procedures.
2. Patients admitted preoperatively to neurology or neurological intensive care units (neuro-ICUs).
3. Patients with ASA classification IV–V or classified as critically ill.
4. Patients with missing or incomplete clinical/laboratory data.

**FIGURE 1:**
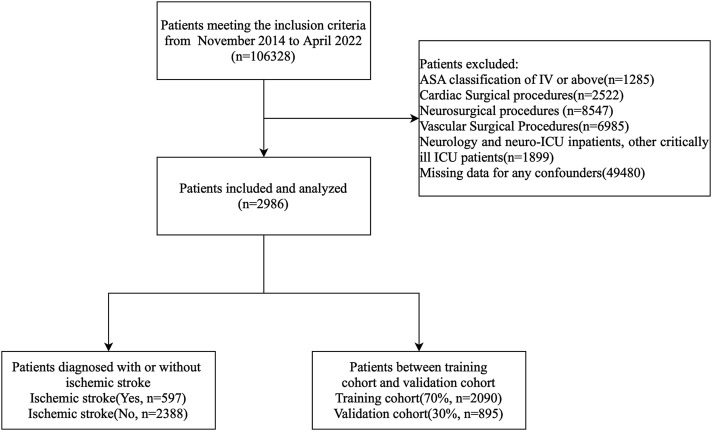
Patient flow diagram. ASA, American Society of Anesthesiologists.

Of the 106,328 patients who underwent noncardiac, nonvascular, and nonneurosurgical surgeries, only those meeting the inclusion criteria were enrolled. A flowchart of the study is presented in Figure 1.

### 2.2 Data collection

#### 2.2.1 Definition of perioperative stroke

The primary outcome was perioperative ischemic stroke, defined as a clinically confirmed cerebral infarction occurring within 30 days postoperatively. Diagnosis was confirmed through imaging (CT/MRI) and neurologist evaluation (Mashour et al., 2011). Diagnostic details were extracted from the electronic medical records (Furie et al., 2011).

#### 2.2.2 Clinical and perioperative parameters

The analysis included clinical data from 2,986 patients who underwent noncardiac, nonvascular, and nonneurosurgical procedures. Comprehensive demographic, clinical, imaging, and laboratory parameters were systematically collected from the preoperative period through 7 days postoperatively.

#### 2.2.3 The key variables included the following

1. Demographics: Age, sex, height, weight, and BMI.
2. Surgical Parameters: ASA classification, procedure type and duration, anesthesia duration and method, urine output, and blood loss.
3. Comorbidities: Smoking, alcohol use, ascites, hypertension, diabetes, cardiac disorders, COPD, renal disease, and cerebrovascular disease.
4. Laboratory Tests: Complete blood count; creatinine, albumin, and liver enzyme levels; and coagulation profiles.
5. Preoperative Medications: Antihypertensives, anticoagulants, and antiplatelet agents, including documentation of discontinuation timelines.
6. Intraoperative Monitoring: Blood pressure, heart rate, temperature, bispectral index (BIS), and end-tidal CO_2_, recorded at 5-min intervals.
7. Intraoperative Medications: Inhalational anesthetics, diuretics, and anticoagulants.
8. Intraoperative Fluids/Transfusions: Colloids, crystalloids, and blood products.
9. Vasopressors: Ephedrine and phenylephrine.
10. Postoperative Medications: Statins, anticoagulants, and antiplatelet agents (dosage and timing recorded) administered within 7 days to prevent secondary cerebrovascular events.

### 2.3 Data preprocessing

The data preprocessing and model development for this study involved feature selection, multiple imputation, and standardization (Bellini et al., 2022). Variables were initially excluded based on two criteria: (1) missing values exceeding 20% (Austin et al., 2021) and (2) lack of established associations with perioperative stroke, as supported by the literature and clinical expertise (Mashour et al., 2011). After screening, 22 variables were retained: emergency surgery, angina, valvular heart disease, stroke history, tumor history, intraoperative mean arterial pressure ≤75 mmHg, preoperative use of metoprolol/diuretics/insulin, intraoperative use of remifentanil, age, surgical duration, preoperative red blood cell/lymphocyte/basophil counts, mean corpuscular volume, hematocrit, total protein, activated partial thromboplastin time (APTT), prothrombin time (PT), fibrinogen, and succinylated gelatine administration (Bijker et al., 2012).

Residual missing values were imputed using the R-based MICE (Multiple Imputation by Chained Equations) package (Ren et al., 2023). After imputation, all variables were Z-score normalized to standardize data scales and minimize overfitting.

### 2.4 Propensity score matching (PSM)

To minimize selection bias and create a balanced control group, propensity score matching (PSM) was performed. Propensity scores were estimated using key demographic and clinical variables, including age, sex, comorbidities (e.g., hypertension, diabetes, stroke history), and other factors known to influence perioperative stroke risk. These variables were chosen based on their clinical relevance, established associations with stroke risk in the literature, and expert consensus.

The matching procedure utilized the nearest neighbor matching algorithm with a 1:4 ratio, where each patient in the stroke group was matched with four controls from the non-stroke group based on the closest propensity score. No caliper was applied, although using one could have improved match quality by restricting matches to those with more similar propensity scores.

After matching, the balance between the stroke and non-stroke groups was assessed using standardized mean differences (SMDs). Covariates with an SMD of less than 0.1 were considered well-balanced. This approach aimed to reduce confounding and ensure comparability between the two groups.

**FIGURE 2:**
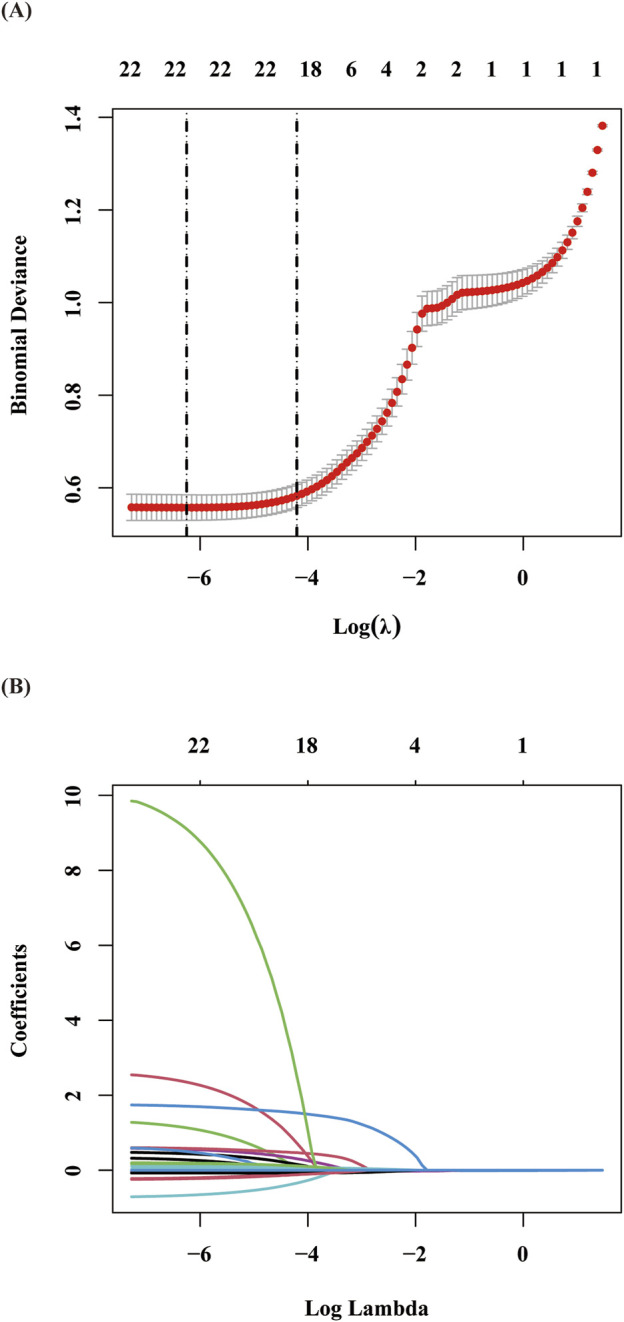
Presentation of the results of the LASSO regression analysis: **(A)** Variable selection in the LASSO regression model: The left dashed vertical line indicates the optimal lambda value (lambda⋅min) corresponding to the minimum cross-validated error, while the right dashed vertical line represents the largest lambda value within one standard error of the optimal value (lambda.1se). **(B)** Coefficient shrinkage patterns of the LASSO regression model: The trajectories illustrate the coefficient paths for all candidate variables across decreasing lambda values, demonstrating the feature selection process through progressive regularization.

The final cohort included 597 stroke patients and 2,388 non-stroke controls. Given the balanced group proportions, no oversampling was needed to avoid prediction bias. The data were randomly split into training (70%) and validation (30%) sets. LASSO regression identified 11 key predictors, which were validated through 10-fold cross-validation (Staartjes et al., 2022) (Figure 2). These predictors included age, stroke history, succinylated gelatine, preoperative APTT, hematocrit, basophil count, total protein, hypotension, fibrinogen, surgical duration, and preoperative insulin.

### 2.5 Machine learning model development

A total of nine machine learning algorithms were developed to predict perioperative stroke risk: logistic regression (LR), support vector machine (SVM), gradient boosting machine (GBM), neural network, extreme gradient boosting (XGBoost), k-nearest neighbors (KNN), adaptive boosting (AdaBoost), light gradient boosting machine (LightGBM), and categorical boosting (CatBoost) (Lundberg et al., 2020). The hyperparameters were optimized using grid search, and model robustness was enhanced through 10-fold cross-validation and resampling techniques (Collins et al., 2015).

Model performance was evaluated in three key areas: discrimination, calibration, and clinical utility. Discriminatory power was assessed by the area under the receiver operating characteristic curve (AUC). Calibration was examined using calibration plots and Brier scores. Decision curve analysis (DCA) was used to evaluate the net clinical benefit across varying decision thresholds (Steyerberg and Vergouwe, 2014). Additional performance metrics, including accuracy, sensitivity, specificity, precision, and F1 score, were derived from confusion matrices.

#### 2.5.1 The optimal parameters for each model are as follows

1. Logistic Regression (LR): C = 10, penalty = l2, solver = saga
2. Support Vector Machine (SVM): sigma = 0.001, C = 0.09
3. Gradient Boosting Machine (GBM): n.trees = 100, interaction.depth = 5, shrinkage = 0.1, n.minobsinnode = 30
4. Multi-Layer Perceptron (MLP): size = 6, decay = 0.6
5. XGBoost (XGB): nrounds = 10, max_depth = 3, eta = 0.001, gamma = 0.5, colsample_bytree = 0.5, min_child_weight = 1, subsample = 0.6
6. K-Nearest Neighbors (KNN): kmax = 12, distance = 1, kernel = optimal
7. AdaBoost: mfinal = 2, maxdepth = 2, coeflearn = “Zhu”
8. LightGBM: objective = binary, metric = auc, learning_rate = 1.0, num_threads = 2
9. CatBoost: iterations = 100, eval_metric = AUC, learning_rate = 0.03, random_seed = 123

### 2.6 Model interpretation and visualization

To enhance model interpretability and clinical transparency, SHapley Additive exPlanations (SHAP) analysis was conducted to assess the contribution of each feature to individual predictions. A higher SHAP value indicates a greater influence of the variable on stroke risk estimation (Lundberg et al., 2020).

### 2.7 Statistical analysis

Continuous variables are presented as medians with interquartile ranges and compared using either t-tests or Mann–Whitney U tests, depending on their distribution. Categorical variables are reported as frequencies (percentages) and analyzed with the chi-square test or Fisher’s exact test. Statistical significance was set at *P* < 0.05. All analyses were performed using R version 4.3.0. Model performance metrics included accuracy, sensitivity, precision, specificity, F1 score, and AUC.

## 3 Results

### 3.1 Baseline patient characteristics

As shown in Figure 1, a total of 597 patients with confirmed perioperative stroke were included in the stroke group. To minimize selection bias and ensure a balanced control group, propensity score matching (PSM) was performed using the nearest neighbor matching algorithm at a 1:4 ratio. This was based on key demographic and clinical variables, including age, sex, and comorbidities, which were selected based on clinical relevance and previous studies linking them to perioperative stroke risk. This matching resulted in 2,388 controls for comparative analysis.

Balance between the stroke and non-stroke groups was assessed using standardized mean differences (SMDs). Covariates with an SMD of less than 0.1 were considered well-balanced after matching. The data were then divided into model development (n = 2,090, 70%) and validation (n = 895, 30%) sets using stratified random sampling. Table 1 presents the baseline characteristics of stroke and non-stroke patients, while Table 2 outlines the intergroup differences between the training and validation sets.

**TABLE 1:** Baseline demographic and clinical characteristics of included patients diagnosed with or without ischemic stroke.

Characteristics	Total	No postoperative ischemic stroke N = 2,388	Postoperative ischemic stroke N = 597	*P*
Emergency surgery, n (%)				<0.001
No	2,564 (86%)	2,094 (88%)	470 (79%)	
Yes	421 (14%)	294 (12%)	127 (21%)	
Sex, n (%)				0.003
male	1,351 (45%)	1,048 (44%)	303 (51%)	
female	1,634 (55%)	1,340 (56%)	294 (49%)	
Age, years	56 (44, 68)	52 (40, 63)	71 (66, 75)	<0.001
Surgery length, min	143 (100, 210)	145 (100, 217)	136 (106, 198)	0.8
ASA classification, n (%)				<0.001
I	224 (7.5%)	224 (9.4%)	0 (0%)	
II	2,132 (71%)	1,814 (76%)	318 (53%)	
III	629 (21%)	350 (15%)	279 (47%)	
Amount of blood loss, ml	50 (20, 200)	50 (20, 200)	93 (48, 189)	<0.001
Hypertension, n (%)				<0.001
No	2,471 (83%)	2,089 (87%)	382 (64%)	
Yes	514 (17%)	299 (13%)	215 (36%)	
Diabetes, n (%)				<0.001
No	2,735 (92%)	2,230 (93%)	505 (85%)	
Yes	250 (8.4%)	158 (6.6%)	92 (15%)	
Coronary heart disease, n (%)				0.006
No	2,844 (95%)	2,288 (96%)	556 (93%)	
Yes	141 (4.7%)	100 (4.2%)	41 (6.9%)	
Angina pectoris, n (%)				<0.001
No	2,969 (99%)	2,384 (100%)	585 (98%)	
Yes	16 (0.5%)	4 (0.2%)	12 (2.0%)	
Valvular heart disease, n (%)				<0.001
No	2,923 (98%)	2,372 (99%)	551 (92%)	
Yes	62 (2.1%)	16 (0.7%)	46 (7.7%)	
Myocardial infarction, n (%)				<0.001
No	2,969 (99%)	2,382 (100%)	587 (98%)	
Yes	16 (0.5%)	6 (0.3%)	10 (1.7%)	
Heart failure, n (%)				0.017
No	2,979 (100%)	2,386 (100%)	593 (99%)	
Yes	6 (0.2%)	2 (<0.1%)	4 (0.7%)	
Atrial fibrillation, n (%)				0.085
No	2,976 (100%)	2,383 (100%)	593 (99%)	
Yes	9 (0.3%)	5 (0.2%)	4 (0.7%)	
Peripheral vascular disease, n (%)				<0.001
No	2,678 (90%)	2,243 (94%)	435 (73%)	
Yes	307 (10%)	145 (6.1%)	162 (27%)	
Renal insufficiency, n (%)				0.14
No	2,973 (100%)	2,376 (99%)	597 (100%)	
Yes	12 (0.4%)	12 (0.5%)	0 (0%)	
Previous stroke, n (%)				<0.001
No	2,729 (91%)	2,320 (97%)	409 (69%)	
Yes	256 (8.6%)	68 (2.8%)	188 (31%)	
Malignant tumor, n (%)				<0.001
No	2,658 (89%)	2,097 (88%)	561 (94%)	
Yes	327 (11%)	291 (12%)	36 (6.0%)	
Preoperative hemoglobin, g/L	125 (112, 137)	127 (113, 139)	119 (108, 131)	<0.001
Preoperative serum albumin, g/L	39.6 (36.2, 43.4)	40.6 (37.0, 44.0)	36.8 (34.2, 39.1)	<0.001
Preoperative total bilirubin, μmol/L	10 (8, 15)	10 (8, 14)	11 (8, 15)	0.02
Preoperative thrombin time’s	16.70 (15.70, 17.70)	16.50 (15.50, 17.60)	17.10 (16.30, 17.87)	<0.001
Preoperative ACEI drugs, n (%)				<0.001
No	2,791 (94%)	2,252 (94%)	539 (90%)	
Yes	194 (6.5%)	136 (5.7%)	58 (9.7%)	
Preoperative ARB drugs, n (%)				0.002
No	2,838 (95%)	2,285 (96%)	553 (93%)	
Yes	147 (4.9%)	103 (4.3%)	44 (7.4%)	
Preoperative steroids, n (%)				0.2
No	2,395 (80%)	1,928 (81%)	467 (78%)	
Yes	590 (20%)	460 (19%)	130 (22%)	
Preoperative β-blockers, n (%)				<0.001
No	2,770 (93%)	2,283 (96%)	487 (82%)	
Yes	215 (7.2%)	105 (4.4%)	110 (18%)	
Preoperative calcium channel blockers, n (%)				<0.001
No	2,462 (82%)	2,105 (88%)	357 (60%)	
Yes	523 (18%)	283 (12%)	240 (40%)	
Perioperative nonsteroidal drugs, n (%)				<0.001
No	507 (17%)	493 (21%)	14 (2.3%)	
Yes	2,478 (83%)	1,895 (79%)	583 (98%)	
Colloids, mL	500 (0, 500)	500 (0, 500)	383 (85, 500)	0.013
Crystals, mL	1,500 (1,000, 2,000)	1,500 (1,000, 2,000)	1,500 (1,191, 1,912)	0.018
Blood product usage, n (%)				<0.001
No	2,511 (84%)	2,105 (88%)	406 (68%)	
Yes	474 (16%)	283 (12%)	191 (32%)	
Intraoperative steroids, n (%)				<0.001
No	2,336 (78%)	1,957 (82%)	379 (63%)	
Yes	649 (22%)	431 (18%)	218 (37%)	

P-values were determined using χ^2^ or Fisher’s exact tests for categorical variables and analysis of variance or Kruskal–Wallis tests for continuous variables. Categorical data were reported as frequencies (percentages), and continuous variables were reported as medians (quartiles). ACEIs, angiotensin-converting enzyme inhibitors; ARBs, angiotensin II, receptor blockers; ASA, american society of anesthesiologists; BMI, body mass index; MAP, mean arterial pressure.

**TABLE 2:** Baseline demographic and clinical characteristics of included patients between training and validation sets.

Characteristics	Total	Training sets n = 2,090	Validation sets n = 895	*P*
Ischemic stroke, n (%)				>0.9
No	2,388 (80%)	1,672 (80%)	716 (80%)	
Yes	597 (20%)	418 (20%)	179 (20%)	
Emergency surgery, n (%)				0.8
No	2,564 (86%)	1,797 (86%)	767 (86%)	
Yes	421 (14%)	293 (14%)	128 (14%)	
Sex, n (%)				0.2
male	1,351 (45%)	931 (45%)	420 (47%)	
female	1,634 (55%)	1,159 (55%)	475 (53%)	
Age, (Median [Q1, Q3]), yr	56 (44, 68)	56 (43, 68)	56 (44, 68)	>0.9
Surgery length, (Median [Q1, Q3]), min	143 (100, 210)	143 (100, 212)	144 (100, 210)	0.6
ASA classification, n (%)				0.8
I	224 (7.5%)	153 (7.3%)	71 (7.9%)	
II	2,132 (71%)	1,499 (72%)	633 (71%)	
III	629 (21%)	438 (21%)	191 (21%)	
Amount of blood loss, ml	50 (20, 200)	50 (20, 200)	50 (20, 182)	0.2
Hypertension, n (%)				0.3
No	2,471 (83%)	1,740 (83%)	731 (82%)	
Yes	514 (17%)	350 (17%)	164 (18%)	
Diabetes, n (%)				0.5
No	2,735 (92%)	1,920 (92%)	815 (91%)	
Yes	250 (8.4%)	170 (8.1%)	80 (8.9%)	
Coronary heart disease, n (%)				0.15
No	2,844 (95%)	1,999 (96%)	845 (94%)	
Yes	141 (4.7%)	91 (4.4%)	50 (5.6%)	
Angina pectoris, n (%)				0.6
No	2,969 (99%)	2,080 (100%)	889 (99%)	
Yes	16 (0.5%)	10 (0.5%)	6 (0.7%)	
Valvular heart disease, n (%)				0.7
No	2,923 (98%)	2,048 (98%)	875 (98%)	
Yes	62 (2.1%)	42 (2.0%)	20 (2.2%)	
Myocardial infarction, n (%)				0.051
No	2,969 (99%)	2,075 (99%)	894 (100%)	
Yes	16 (0.5%)	15 (0.7%)	1 (0.1%)	
Heart failure, n (%)				0.2
No	2,979 (100%)	2,084 (100%)	895 (100%)	
Yes	6 (0.2%)	6 (0.3%)	0 (0%)	
Atrial fibrillation, n (%)				0.7
No	2,976 (100%)	2,084 (100%)	892 (100%)	
Yes	9 (0.3%)	6 (0.3%)	3 (0.3%)	
Peripheral vascular disease, n (%)				>0.9
No	2,678 (90%)	1,875 (90%)	803 (90%)	
Yes	307 (10%)	215 (10%)	92 (10%)	
Renal insufficiency, n (%)				>0.9
No	2,973 (100%)	2,081 (100%)	892 (100%)	
Yes	12 (0.4%)	9 (0.4%)	3 (0.3%)	
Previous stroke, n (%)				0.5
No	2,729 (91%)	1,906 (91%)	823 (92%)	
Yes	256 (8.6%)	184 (8.8%)	72 (8.0%)	
Malignant tumor, n (%)				0.4
No	2,658 (89%)	1,854 (89%)	804 (90%)	
Yes	327 (11%)	236 (11%)	91 (10%)	
Preoperative hemoglobin, (Median [Q1, Q3]), g/L	125 (112, 137)	125 (112, 137)	125 (111, 137)	>0.9
Preoperative serum albumin, (Median [Q1, Q3]), g/L	39.6 (36.2, 43.4)	39.6 (36.2, 43.4)	39.6 (36.2, 43.3)	0.9
Preoperative total bilirubin, (Median [Q1, Q3]), μmol/L	10 (8, 15)	10 (8, 15)	10 (8, 15)	>0.9
Preoperative thrombin time, (Median [Q1, Q3]), s	16.70 (15.70, 17.70)	16.70 (15.70, 17.70)	16.74 (15.70, 17.83)	0.1
Preoperative ACEI drugs, n (%)				0.2
No	2,791 (94%)	1,963 (94%)	828 (93%)	
Yes	194 (6.5%)	127 (6.1%)	67 (7.5%)	
Preoperative ARB drugs, n (%)				0.14
No	2,838 (95%)	1,979 (95%)	859 (96%)	
Yes	147 (4.9%)	111 (5.3%)	36 (4.0%)	
Preoperative steroids, n (%)				0.3
No	2,395 (80%)	1,666 (80%)	729 (81%)	
Yes	590 (20%)	424 (20%)	166 (19%)	
Preoperative β-blockers, n (%)				0.8
No	2,770 (93%)	1,941 (93%)	829 (93%)	
Yes	215 (7.2%)	149 (7.1%)	66 (7.4%)	
Preoperative calcium channel blockers, n (%)				0.7
No	2,462 (82%)	1,728 (83%)	734 (82%)	
Yes	523 (18%)	362 (17%)	161 (18%)	
Perioperative nonsteroidal drugs, n (%)				0.2
No	507 (17%)	368 (18%)	139 (16%)	
Yes	2,478 (83%)	1,722 (82%)	756 (84%)	
Colloids, mL	500 (0, 500)	500 (0, 500)	500 (0, 500)	0.7
Crystals, mL	1,500 (1,000, 2,000)	1,500 (1,000, 2,000)	1,500 (1,000, 2,000)	0.7
Blood product usage, n (%)				0.8
No	2,511 (84%)	1,756 (84%)	755 (84%)	
Yes	474 (16%)	334 (16%)	140 (16%)	
Intraoperative steroids, n (%)				0.4
No	2,336 (78%)	1,626 (78%)	710 (79%)	
Yes	649 (22%)	464 (22%)	185 (21%)	

P-values were determined using χ^2^ or Fisher’s exact tests for categorical variables and analysis of variance or Kruskal–Wallis tests for continuous variables. Categorical data were reported as frequencies (percentages), and continuous variables were reported as medians (quartiles). ACEIs, angiotensin-converting enzyme inhibitors; ARBs, angiotensin II, receptor blockers; ASA, american society of anesthesiologists; BMI, body mass index; MAP, mean arterial pressure.

### 3.2 Performance evaluation of the nine models

**FIGURE 3:**
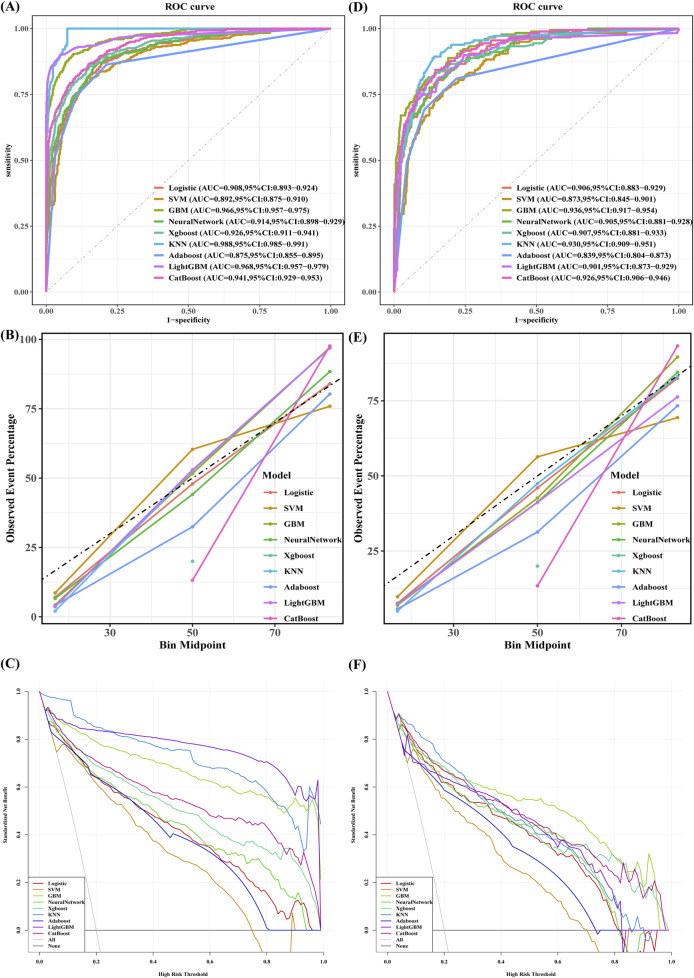
The performance and comparison of nine different predictive models: **(A)** ROC Curves for the Training sets; **(B)** Calibration Curve for the Training sets; **(C)** Decision Curve Analysis for the Training sets; **(D)** ROC Curves for the Validation sets; **(E)** Calibration Curve for the Validation sets; **(F)** Decision Curve Analysis for the Validation sets.

Nine machine learning models were developed to predict stroke risk in noncardiac, nonvascular, and nonneurosurgical patients. Figure 3 illustrates the model performance metrics, including receiver operating characteristic (ROC) curves, calibration plots, and decision curve analysis (DCA), for both the training and validation sets.

The gradient boosting machine (GBM) model demonstrated the best overall predictive performance. In the training set, GBM achieved an AUC of 0.966 (95% CI: 0.957–0.975), with an accuracy of 90.4%, sensitivity of 90.4%, precision of 70.1%, specificity of 81.8%, and an F1 score of 79.0%. Its performance remained robust on the validation set, with an AUC of 0.936 (95% CI: 0.917–0.954), accuracy of 82.6%, sensitivity of 88.8%, precision of 53.9%, specificity of 81.0%, and an F1 score of 67.1%.

In contrast, the k-nearest neighbors (KNN) algorithm achieved the highest training AUC of 0.988 (Figure 3A), but its performance was less generalizable due to overfitting, as reflected in its lower performance on the validation set. Specifically, KNN had an accuracy of 86.6%, sensitivity of 89.4%, specificity of 85.9%, precision of 61.3%, an F1 score of 72.7%, and an AUC of 0.930.

Similarly, CatBoost demonstrated competitive performance with a validation AUC of 0.926 (Figure 3D). Its accuracy was 85.3%, sensitivity 84.4%, specificity 85.5%, precision 59.2%, F1 score 69.6%, and AUC 0.926.

Although KNN showed the highest training AUC, GBM was ultimately selected as the optimal model due to its consistent performance across both the training and validation sets. GBM outperformed KNN on the validation set, achieving a higher AUC and F1 score, and providing a better balance of sensitivity, specificity, and precision. Despite CatBoost showing competitive results, particularly in sensitivity and specificity, it was still outperformed by GBM in accuracy, sensitivity, and AUC.

In conclusion, GBM demonstrated superior overall performance, consistently outperforming KNN, CatBoost, and other models, including logistic regression, neural networks, XGBoost, and LightGBM (Table 3). Consequently, it was selected as the optimal model for predicting perioperative stroke risk.

**TABLE 3:** Performance metrics of various machine learning models for predicting perioperative ischemic stroke risk in patients across training and validation sets.

Dataset	Evaluation Metric	Logistic	SVM	GBM	NeuralNetwork	Xgboost	KNN	Adaboost	LightGBM	CatBoost
Training	Accuracy	0.817	0.855	0.904	0.829	0.849	0.941	0.803	0.941	0.854
	Sensitivity	0.871	0.785	0.904	0.871	0.856	1	0.864	0.897	0.866
	Precision	0.525	0.606	0.701	0.545	0.584	0.771	0.504	0.822	0.592
	Specificity	0.803	0.873	0.904	0.818	0.847	0.926	0.788	0.952	0.851
	F1 Score	0.655	0.684	0.79	0.67	0.694	0.871	0.637	0.858	0.704
	AUC	0.908	0.892	0.966	0.914	0.926	0.988	0.875	0.968	0.941
Validation	Accuracy	0.822	0.815	0.826	0.836	0.863	0.866	0.787	0.836	0.853
	Sensitivity	0.838	0.765	0.888	0.81	0.816	0.894	0.81	0.816	0.844
	Precision	0.536	0.525	0.539	0.562	0.619	0.613	0.48	0.562	0.592
	Specificity	0.818	0.827	0.81	0.842	0.874	0.859	0.781	0.841	0.855
	F1 Score	0.654	0.623	0.671	0.664	0.704	0.727	0.603	0.665	0.696
	AUC	0.906	0.873	0.936	0.905	0.907	0.93	0.839	0.901	0.926

### 3.3 Model interpretability and visualization

**FIGURE 4:**
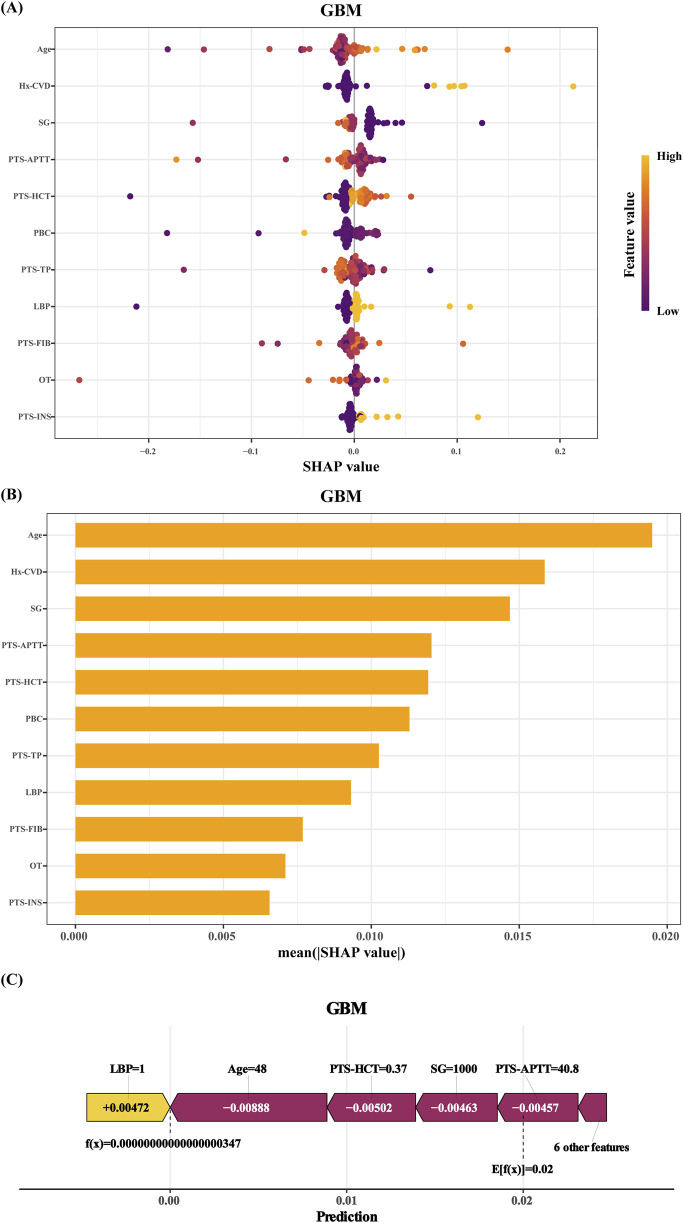
SHAP of the model: **(A)** Characteristic attributes in SHAP. The abscissa is the SHAP value, and each line denotes a feature. Higher eigenvalues are indicated by purple dots, and lower eigenvalues are indicated by yellow dots; **(B)** Feature importance ranking of the Gradient Boosting Machine (GBM) model; **(C)** Interpretability analysis of 1 independent samples. Hx-CVD:a preoperative history of stroke. SG: succinylated gelatin. PTS-APTT: preoperative APTT. PTS-HCT: preoperative hematocrit. PBC: preoperative basophil count. PTS-TP: preoperative total protein. LBP: hypotension. PTS-FIB: preoperative fibrinogen. OT: operative time. PTS-INS: preoperative insulin.

Figure 4A presents the SHAP (SHapley Additive exPlanations) summary plot for GBM, where the X-axis represents SHAP values (higher values indicate stronger contributions to stroke prediction). Feature magnitude is represented by a color gradient, ranging from purple (high values) to yellow (low values).

The top four predictors of stroke risk were age, stroke history, succinylated gelatine dosage, and preoperative activated partial thromboplastin time (APTT). Older age, a prior stroke history, higher succinylated gelatine use, and lower preoperative APTT were strongly associated with an increased stroke risk. Other significant factors included reduced haematocrit, lower basophil count, elevated total protein, intraoperative hypotension (mean arterial pressure ≤75 mmHg for ≥5 min), elevated fibrinogen levels, longer surgical duration, and infrequent preoperative insulin use.

These findings were further supported by the feature importance rankings in Figure 4B. The interpretability of the model was also demonstrated through individualized case analyses. Figure 4C shows SHAP force plots for a representative non-stroke patient, highlighting feature-specific contributions to the prediction.

**FIGURE 5:**
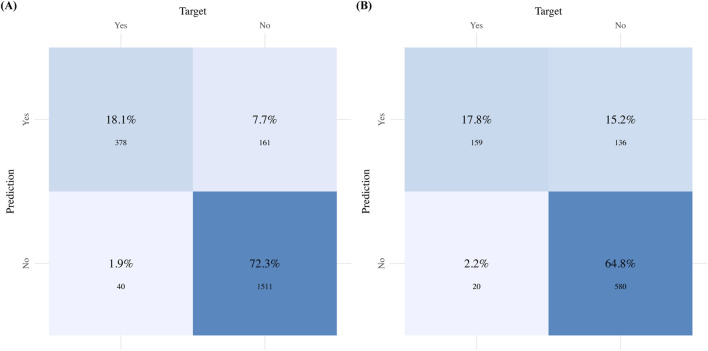
Confusion matrix for the GBM model: **(A)** Confusion matrix for the Training sets; **(B)** Confusion matrix for the Validation sets.

**FIGURE 6:**
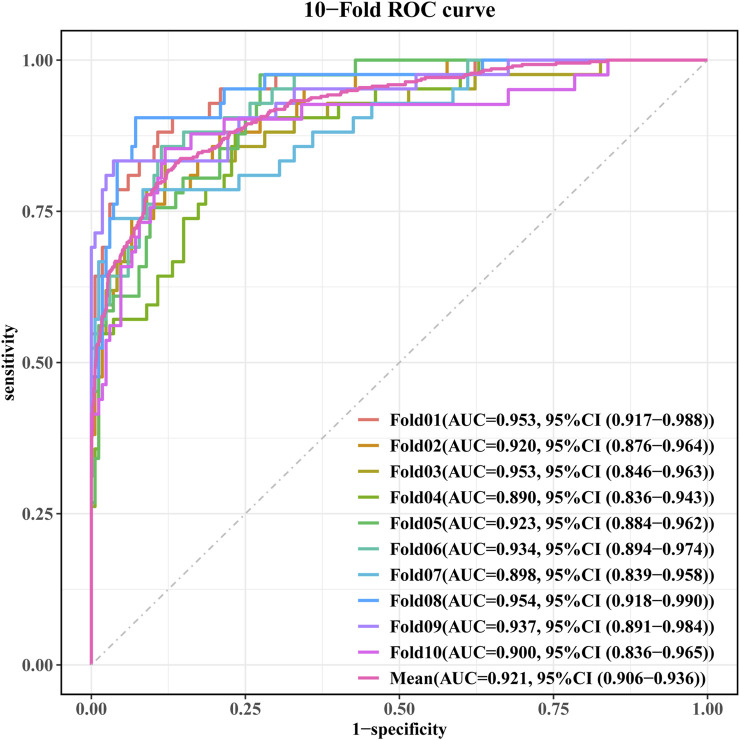
Performance metrics of the GBM model across the 10-fold cross-validation.

Figures 5A,B complement these statistical findings by presenting the confusion matrix for the GBM model, showing both actual and predicted values. Finally, Figure 6 presents the performance metrics of the GBM model across the 10-fold cross-validation.

## 4 Discussion

This study developed a perioperative stroke risk prediction model for patients undergoing noncardiac, nonvascular, and nonneurosurgical procedures. By integrating multiple machine learning algorithms with a large-scale perioperative database from Henan Provincial People’s Hospital, we identified 16 key factors contributing to perioperative stroke risk prediction (Bilimoria et al., 2013). While traditional perioperative stroke assessment tools, such as the National Institutes of Health Stroke Scale (NIHSS), modified Rankin Scale (mRS), and ABCD2 score, are useful for preliminary risk identification, they have limitations in predicting future stroke incidence (Birman-Deych et al., 2005; Sacco et al., 2013). This study is the first to create a prediction model specifically designed for Chinese surgical patients undergoing noncardiac, nonvascular, and nonneurosurgical procedures. It systematically integrates standardized demographic data, comorbidities, and health-related indicators, providing significant innovation. The model’s strength lies in its ability to reveal complex interactions among high-dimensional variables through machine learning, leading to enhanced predictive accuracy and offering robust evidence for stroke prediction in this surgical cohort (Deo, 2015).

Through LASSO regression analysis, we identified 11 key predictive variables. As shown in Figure 2, advanced age, a history of stroke, increased succinylated gelatine administration, and lower preoperative activated partial thromboplastin time (APTT) were significantly associated with increased stroke risk. Other independent predictors included reduced haematocrit, decreased preoperative basophil count, elevated preoperative total protein, mean arterial pressure ≤75 mmHg for ≥5 min, elevated preoperative fibrinogen levels, prolonged surgical duration, and infrequent preoperative insulin use.

These findings align with existing literature. Advanced age and a prior history of stroke are well-established predictors of perioperative stroke risk (Mashour et al., 2011). Age-related changes such as vascular aging and arteriosclerosis impair cerebral perfusion (Fanning et al., 2024), increasing cerebrovascular fragility and stroke susceptibility. Older patients with a history of cerebrovascular events exhibit significantly higher rates of postoperative stroke recurrence than those without such history (Vlisides and Mashour, 2016). Additionally, the frequent coexistence of chronic comorbidities such as hypertension, diabetes, and atherosclerosis in older populations further exacerbates perioperative stroke risk through shared pathological mechanisms (Sultan et al., 2020). These findings underscore the importance of thorough preoperative risk stratification and tailored interventions for high-risk groups.

Coagulation abnormalities are another critical determinant of perioperative stroke risk. The dose-dependent relationship between succinylated gelatine administration (commonly used in hepatectomy fluid management) and postoperative coagulation dysfunction can predispose individuals to stroke (Kuang et al., 2024). Experimental evidence shows that gelatine inhibits platelet aggregation, particularly at doses exceeding 20 mL/kg (Cai et al., 2021), and induces endothelial cell activation and hypercoagulable states with prolonged administration (Malerba et al., 2017). Similarly, preoperative hypocoagulable profiles (indicated by reduced APTT) and elevated fibrinogen levels serve as markers of prothrombotic tendencies, promoting thrombogenesis and cerebrovascular events (Li et al., 2022; Alemayehu et al., 2024). These findings highlight the importance of preoperative coagulation profiling and evidence-based corrective strategies to mitigate perioperative stroke risk.

Perioperative haemodynamic fluctuations, surgical duration, and insulin management were also identified as key stroke risk factors. Intraoperative hypotension, a major contributor to cerebral hypoperfusion, impairs cerebral blood flow and increases susceptibility to ischemic stroke (Mazzeffi et al., 2021; Wongtangman et al., 2021). Prolonged surgery duration increases exposure to physiological stress, elevating the risk of postoperative complications, including cerebrovascular events (Mazzeffi et al., 2021). Among diabetic patients, those with suboptimal insulin use or poor glycemic control have a significantly higher likelihood of postoperative stroke compared to those with good glycemic control (Eshuis et al., 2011; Sacco et al., 2024). These findings emphasize the need for maintaining haemodynamic stability, optimizing glycemic control, and managing intraoperative blood pressure as essential strategies for reducing stroke risk.

In terms of model performance, the gradient boosting machine (GBM) outperformed other models in the validation cohort, achieving superior AUC values, accuracy, specificity, sensitivity, and F1-score metrics. Decision curve analysis (DCA) demonstrated substantial net clinical benefits for the GBM model across most threshold probabilities, except in highly risk-averse scenarios (Figures 3C,F). Calibration analysis produced Brier scores of 0.05 (95% CI: 0.045–0.057) for the validation sets and 0.072 (95% CI: 0.06–0.085) for the test sets, indicating excellent alignment between predicted probabilities and observed outcomes. A comprehensive evaluation confirmed the robustness and generalizability of the GBM, establishing it as the optimal model. Its computational efficiency, adaptability to complex scenarios, and ability to handle high-dimensional nonlinear relationships affirm its superiority in medical data analytics (Langenberger et al., 2023).

Despite the widespread use of logistic regression in clinical research, its application in perioperative stroke prediction has notable limitations. First, logistic regression’s linearity assumption restricts its predictive power, failing to account for complex interactions and nonlinear associations, thus compromising prediction accuracy (Nusinovici et al., 2020; Song et al., 2023). Second, these models struggle with dynamic data integration, particularly real-time intraoperative monitoring parameters and time-dependent variables (e.g., the interaction between anticoagulant withdrawal duration and surgical timing) (Klug et al., 2024). Additionally, logistic regression models rely heavily on manual feature engineering, risking the omission of critical predictors, such as intraoperative micro-embolic signals or postoperative fluctuations in the neutrophil-to-lymphocyte ratio (NLR) (Zhuang et al., 2021; Oh et al., 2024). Emerging evidence suggests that these dynamic indicators could improve predictive performance by up to 15% (Zhuang et al., 2021).

Moreover, logistic regression is highly sensitive to class imbalance, often requiring remedial techniques like oversampling, which can introduce prediction bias (van den Goorbergh et al., 2022). More importantly, the model lacks the ability to dynamically recalibrate risk, preventing real-time prediction adjustments (Xue et al., 2022). Studies suggest this limitation may lead to the misclassification of up to 38% of high-risk patients (Cooper et al., 2016). These limitations make logistic regression insufficient for modern perioperative stroke prediction in precision medicine (Kim et al., 2023).

This study introduces key advancements in perioperative stroke prediction through the following innovations:

1. Data Integration: We consolidated the perioperative database from Henan Provincial People’s Hospital, integrating preoperative baseline characteristics, intraoperative dynamic monitoring metrics (e.g., haemodynamic fluctuations, heart rate, and oxygen saturation), and postoperative complications. This integration provides comprehensive insights into patients’ physiological status.
2. Cohort Expansion: The analysis included over 10,000 surgical cases, making it the largest cohort in perioperative stroke prediction research. This large-scale approach improves model robustness and generalizability, enhancing clinical applicability.
3. Novel Predictive Framework: We developed a gradient boosting machine (GBM) model capable of processing both structured clinical data and intraoperative continuous monitoring signals. The model showed exceptional discriminative performance (AUC 0.936), outperforming traditional regression models in perioperative stroke prediction.
4. Clinical Decision Support System: A real-time decision support system based on this predictive model was implemented, offering dynamic risk score updates and alerts. Currently being piloted in three tertiary hospitals, the system has received positive feedback for its ability to identify high-risk patients early and facilitate timely interventions.

These innovations collectively improve the accuracy of perioperative stroke prediction and support the clinical implementation of predictive models. The established framework offers a practical solution for optimizing perioperative care.

Although this study represents significant progress, several limitations should be acknowledged. First, the retrospective nature of the analysis, relying on a single data source, may introduce potential selection bias (Oh et al., 2024). Second, the absence of key stroke-related variables such as genetic profiles and socioeconomic factors limits the comprehensiveness of the risk assessment. Finally, as a retrospective cohort study, the lack of prospective follow-up data for disease progression may introduce confounding factors. Future studies should incorporate updated datasets to further refine and validate the model.

Despite these limitations, this research addresses a critical gap in perioperative stroke prediction, using advanced algorithms like the gradient boosting machine (GBM) to enhance predictive accuracy. The web-based prediction platform developed offers considerable clinical potential for rapid high-risk patient identification and personalized interventions (Teng et al., 2024). This tool also empowers patients with self-assessment capabilities, improving disease awareness and management.

## 5 Conclusion

This study developed a machine learning-based model for perioperative stroke prediction, demonstrating superior accuracy compared to traditional methods. The gradient boosting machine (GBM) model, leveraging a wide range of clinical data, showed robust performance and generalizability. Furthermore, the integration of a real-time decision support system enhances the model’s clinical utility, aiding in early identification of high-risk patients and enabling personalized interventions.

## Data Availability

The raw data supporting the conclusions of this article will be made available by the authors, without undue reservation.
