# Supplementary material for: Development and validation of a machine learning-based model for perioperative stroke prediction in noncardiac, nonvascular, and nonneurosurgical patients
Source: Front Physiol. 2025 Jun 20;16:1624898. doi: 10.3389/fphys.2025.1624898 (PMC12226270; doi:10.3389/fphys.2025.1624898)
Supplement: Supplementary file 1 [file Table1.docx]

**Table 1** Baseline demographic and clinical characteristics of included patients diagnosed with or without ischemic stroke.

| Characteristics | Total | No postoperative ischemic stroke  N = 2,388 | Postoperative ischemic stroke  N = 597 | *P* |
| --- | --- | --- | --- | --- |
| Emergency surgery, n (%) |  |  |  | <0.001 |
| No | 2,564 (86%) | 2,094 (88%) | 470 (79%) |  |
| Yes | 421 (14%) | 294 (12%) | 127 (21%) |  |
| Sex, n (%) |  |  |  | 0.003 |
| male | 1,351 (45%) | 1,048 (44%) | 303 (51%) |  |
| female | 1,634 (55%) | 1,340 (56%) | 294 (49%) |  |
| Age, years | 56 (44, 68) | 52 (40, 63) | 71 (66, 75) | <0.001 |
| Surgery length, min | 143 (100, 210) | 145 (100, 217) | 136 (106, 198) | 0.8 |
| ASA classification, n (%) |  |  |  | <0.001 |
| I | 224 (7.5%) | 224 (9.4%) | 0 (0%) |  |
| II | 2,132 (71%) | 1,814 (76%) | 318 (53%) |  |
| III | 629 (21%) | 350 (15%) | 279 (47%) |  |
| Amount of blood loss, ml | 50 (20, 200) | 50 (20, 200) | 93 (48, 189) | <0.001 |
| Hypertension, n (%) |  |  |  | <0.001 |
| No | 2,471 (83%) | 2,089 (87%) | 382 (64%) |  |
| Yes | 514 (17%) | 299 (13%) | 215 (36%) |  |
| Diabetes, n (%) |  |  |  | <0.001 |
| No | 2,735 (92%) | 2,230 (93%) | 505 (85%) |  |
| Yes | 250 (8.4%) | 158 (6.6%) | 92 (15%) |  |
| Coronary heart disease, n (%) |  |  |  | 0.006 |
| No | 2,844 (95%) | 2,288 (96%) | 556 (93%) |  |
| Yes | 141 (4.7%) | 100 (4.2%) | 41 (6.9%) |  |
| Angina pectoris, n (%) |  |  |  | <0.001 |
| No | 2,969 (99%) | 2,384 (100%) | 585 (98%) |  |
| Yes | 16 (0.5%) | 4 (0.2%) | 12 (2.0%) |  |
| Valvular heart disease, n (%) |  |  |  | <0.001 |
| No | 2,923 (98%) | 2,372 (99%) | 551 (92%) |  |
| Yes | 62 (2.1%) | 16 (0.7%) | 46 (7.7%) |  |
| Myocardial infarction, n (%) |  |  |  | <0.001 |
| No | 2,969 (99%) | 2,382 (100%) | 587 (98%) |  |
| Yes | 16 (0.5%) | 6 (0.3%) | 10 (1.7%) |  |
| Heart failure, n (%) |  |  |  | 0.017 |
| No | 2,979 (100%) | 2,386 (100%) | 593 (99%) |  |
| Yes | 6 (0.2%) | 2 (<0.1%) | 4 (0.7%) |  |
| Atrial fibrillation, n (%) |  |  |  | 0.085 |
| No | 2,976 (100%) | 2,383 (100%) | 593 (99%) |  |
| Yes | 9 (0.3%) | 5 (0.2%) | 4 (0.7%) |  |
| Peripheral vascular disease, n (%) |  |  |  | <0.001 |
| No | 2,678 (90%) | 2,243 (94%) | 435 (73%) |  |
| Yes | 307 (10%) | 145 (6.1%) | 162 (27%) |  |
| Renal insufficiency, n (%) |  |  |  | 0.14 |
| No | 2,973 (100%) | 2,376 (99%) | 597 (100%) |  |
| Yes | 12 (0.4%) | 12 (0.5%) | 0 (0%) |  |
| Previous stroke, n (%) |  |  |  | <0.001 |
| No | 2,729 (91%) | 2,320 (97%) | 409 (69%) |  |
| Yes | 256 (8.6%) | 68 (2.8%) | 188 (31%) |  |
| Malignant tumor, n (%) |  |  |  | <0.001 |
| No | 2,658 (89%) | 2,097 (88%) | 561 (94%) |  |
| Yes | 327 (11%) | 291 (12%) | 36 (6.0%) |  |
| Preoperative hemoglobin, g/L | 125 (112, 137) | 127 (113, 139) | 119 (108, 131) | <0.001 |
| Preoperative serum albumin, g/L | 39.6 (36.2, 43.4) | 40.6 (37.0, 44.0) | 36.8 (34.2, 39.1) | <0.001 |
| Preoperative total bilirubin, μmol/L | 10 (8, 15) | 10 (8, 14) | 11 (8, 15) | 0.02 |
| Preoperative thrombin time’s | 16.70 (15.70, 17.70) | 16.50 (15.50, 17.60) | 17.10 (16.30, 17.87) | <0.001 |
| Preoperative ACEI drugs, n (%) |  |  |  | <0.001 |
| No | 2,791 (94%) | 2,252 (94%) | 539 (90%) |  |
| Yes | 194 (6.5%) | 136 (5.7%) | 58 (9.7%) |  |
| Preoperative ARB drugs, n (%) |  |  |  | 0.002 |
| No | 2,838 (95%) | 2,285 (96%) | 553 (93%) |  |
| Yes | 147 (4.9%) | 103 (4.3%) | 44 (7.4%) |  |
| Preoperative steroids, n (%) |  |  |  | 0.2 |
| No | 2,395 (80%) | 1,928 (81%) | 467 (78%) |  |
| Yes | 590 (20%) | 460 (19%) | 130 (22%) |  |
| Preoperative β-blockers, n (%) |  |  |  | <0.001 |
| No | 2,770 (93%) | 2,283 (96%) | 487 (82%) |  |
| Yes | 215 (7.2%) | 105 (4.4%) | 110 (18%) |  |
| Preoperative calcium channel blockers, n (%) |  |  |  | <0.001 |
| No | 2,462 (82%) | 2,105 (88%) | 357 (60%) |  |
| Yes | 523 (18%) | 283 (12%) | 240 (40%) |  |
| Perioperative nonsteroidal drugs, n (%) |  |  |  | <0.001 |
| No | 507 (17%) | 493 (21%) | 14 (2.3%) |  |
| Yes | 2,478 (83%) | 1,895 (79%) | 583 (98%) |  |
| Colloids, mL | 500 (0, 500) | 500 (0, 500) | 383 (85, 500) | 0.013 |
| Crystals, mL | 1,500 (1,000, 2,000) | 1,500 (1,000, 2,000) | 1,500 (1,191, 1,912) | 0.018 |
| Blood product usage, n (%) |  |  |  | <0.001 |
| No | 2,511 (84%) | 2,105 (88%) | 406 (68%) |  |
| Yes | 474 (16%) | 283 (12%) | 191 (32%) |  |
| Intraoperative steroids, n (%) |  |  |  | <0.001 |
| No | 2,336 (78%) | 1,957 (82%) | 379 (63%) |  |
| Yes | 649 (22%) | 431 (18%) | 218 (37%) |  |

P-values were determined using χ^2^ or Fisher’s exact tests for categorical variables and analysis of variance or Kruskal–Wallis tests for continuous variables. Categorical data were reported as frequencies (percentages), and continuous variables were reported as medians (quartiles). ACEIs, angiotensin-converting enzyme inhibitors; ARBs, angiotensin II receptor blockers; ASA, American Society of Anesthesiologists; BMI, body mass index; MAP, mean arterial pressure.
